
JOURNAL INFORMATION
==============================
NLM Title Abbreviation: ScientificWorldJournal
Journal ID: ScientificWorldJournal
NLM Journal ID: 101131163
Journal ID: TSWJ

The Scientific World Journal

ISSN: 2356-6140
EISSN: 1537-744X
Publisher: Wiley

ARTICLE INFORMATION
==============================
PMCID: PMC6084631
DOI: 10.1100/tsw.2001.419
Article ID: 754294
Article version: 1
Subjects: Extended Abstract

Gene Transfer of CGRP Prevents Cerebral Vasoconstriction after Subarachnoid Hemorrhage

Heistad Donald D.
Russo Andrew F.
Faraci Frank M.
Sobey Christopher G.
University of Iowa College of Medicine and VA Medical Center, USA
Publication date: 2001
Electronic publication date: 2001 Feb 16
Volume: 1
First page: 27
Last page: 27
Copyright: Copyright © 2001 Donald D. Heistad et al.
Copyright year: 2001
License: This is an open access article distributed under the Creative Commons Attribution License, which permits unrestricted use, distribution, and reproduction in any medium, provided the original work is properly cited.
License URL: https://creativecommons.org/licenses/by/3.0/

==============================
4th International Meeting on Calcitonin Gene-Related Peptide (CGRP)
*TheScientificWorld* (2001) 1(S1), 27
ISSN 1532-2246; DOI 10.1100/tsw.2001.419

### GENE TRANSFER OF CGRP PREVENTS CEREBRAL VASOCONSTRICTION AFTER SUBARACHNOID HEMORRHAGE

Donald D. Heistad, Andrew F. Russo, Frank M. Faraci, and Christopher G. Sobey
University of Iowa College of Medicine and VA Medical Center

Cerebral vasospasm occurs commonly after subarachnoid hemorrhage (SAH), and is the leading cause of morbidity and mortality after SAH. The mechanism of spasm after SAH is unclear, and prevention is ineffective. Our goal is to develop gene therapy to prevent cerebral vasospasm after SAH.

First, we developed a method to deliver genes to intracranial blood vessels. Instead of injecting a vector intravascularly, a recombinant adenoviral vector was injected into CSF through the cisterna magna. Excellent expression was observed around cerebral vessels at the base of the brain. We identified promoter-enhancers which provide expression throughout the duration of risk of vasospasm.

To identify an appropriate gene to transfer, we applied exogenous substances *in vivo* to the basilar artery of rats after SAH. Cerebral vasodilator responses to acetylcholine are impaired after SAH, responses to activation of $K_{ATP}$ by aprikalim are augmented after SAH, and responses to CGRP (which act in part by activation of $K_{ATP}$) are augmented after SAH.

We therefore cloned the preproCGRP gene and constructed a recombinant adenovirus (AdCGRP). In rabbits after SAH, CGRP levels (by RIA) were 400-fold greater ($2.0 \pm 0.3$ nmol/l) after AdCGRP than after a control virus.

Constrictor responses to serotonin of the basilar artery *ex vivo* are exaggerated after SAH, and these constrictor responses were inhibited by AdCGRP *in vivo*. We examined constrictor responses to SAH of the basilar artery in rabbits *in vivo* with angiography; intracisternal injection of AdCGRP after SAH completely prevented cerebral vasoconstriction.

Before this method can be used after SAH in humans, a better vector (which produces little inflammation) will be needed, or we will need to inject a lower dose of virus to reduce the inflammatory response. We have developed two methods to increase efficiency of gene transfer to intracranial vessels, to allow use of a lower dose of virus.

In summary, gene transfer of CGRP to intracranial blood vessels is a promising approach to prevent cerebral vasospasm and stroke after SAH.
